# Editorial: Generative AI and large language models in microbial evolution, resistance mechanisms, and antimicrobial drug discovery

**DOI:** 10.3389/fmicb.2026.1848209

**Published:** 2026-05-15

**Authors:** Fahim Sufi

**Affiliations:** 1 COEUS Institute, New Market, VA, United States; 2 Centre for Trade and Investment (CTI), University of Dhaka, Dhaka, Bangladesh

**Keywords:** antimicrobial resistance, causal microbiome research, generative AI, large language models (LLM), microbial evolution

## 1 Introduction

The convergence of generative artificial intelligence, large language models, and microbiology is beginning to redefine how researchers conceptualize microbial evolution, antimicrobial resistance, host microbe interactions, and therapeutic discovery. This Research Topic was developed to bring together scholarship at this rapidly expanding interface, with particular interest in predictive and generative frameworks that can illuminate resistance mechanisms, support antimicrobial drug discovery, and improve the interpretability and translational value of computational microbiology. Across the contributions collected here, a central message emerges with unusual clarity: artificial intelligence in microbiology cannot be reduced to prediction alone. Its scientific value depends on biological grounding, methodological rigor, interpretability, and an explicit awareness of equity and biosafety.

## 2 Key trajectories emerging from the Research Topic

The four articles published in this Research Topic collectively illustrate a field moving beyond descriptive analytics toward more ambitious forms of inference and intervention. In Beyond just correlation, Khelfaoui, Wang, Meskher, et al. argue that microbiome research must move from association driven prediction toward causally credible and policy relevant evidence. Their review synthesizes advances in causal machine learning and econometric tools, including instrumental variables, difference in differences, Double Machine Learning, Deep Instrumental Variables, directed acyclic graphs, and federated learning. Particularly noteworthy is their insistence that microbiome analytics should generate evidence that is interpretable, scalable, privacy aware, and actionable for clinical and public health decision making. This contribution is important because it frames causal inference not as a technical refinement at the margins, but as a prerequisite for responsible translation.

A second contribution by Sufi advances a conceptual and normative framework for generative AI in antimicrobial resistance research. Rather than treating AI as a neutral computational engine, the article argues that its usefulness in this domain depends on three interdependent imperatives: evolutionary robustness, explainability with biosafety, and data equity. This is an important intervention because antimicrobial resistance is not a static classification problem. It is an evolutionary process shaped by mutation, horizontal gene transfer, ecological pressure, and uneven global surveillance infrastructures. By insisting that AI models should be evolution aware, interpretable, and globally inclusive, this Perspective broadens the debate from technical feasibility to scientific legitimacy and responsible deployment.

A third contribution by Perzon and Ilan expands the discussion into gut microbial ecology and therapeutic personalization. Their review introduces the Constrained Disorder Principle as a framework for understanding microbial diversity not as a quantity to be maximized indiscriminately, but as a dynamic property that must be maintained within biologically meaningful bounds. This conceptualization is especially relevant to AI enabled microbiome medicine because it resists simplistic assumptions about healthy diversity and instead foregrounds bounded variability, adaptation, and individualized thresholds. The article suggests that AI systems informed by this principle may help predict dysbiosis and refine interventions such as probiotics, prebiotics, fecal microbiota transplantation, and dietary modulation. In doing so, it reminds the field that useful intelligence in microbiology must remain sensitive to temporal fluctuation, heterogeneity, and ecological context.

This line of thinking is extended in the later systematic review by Khelfaoui, Wang, Shehata, et al., which directly addresses the reproducibility crisis in causal microbiome research. That article reviews more than 60 peer reviewed studies and emphasizes persistent weaknesses in validation, interpretability, benchmarking, and reporting. Its most consequential contribution is the proposal of STROBE CML, a reporting framework designed to standardize causal machine learning studies in microbiome science. By advocating synthetic benchmarking, biological plausibility checks, federated validation pipelines, and transparent reporting, the article offers a practical roadmap for strengthening reproducibility and comparability across studies. In a field where computational sophistication often advances faster than methodological standardization, this kind of framework is especially timely.

**Figure 1:**
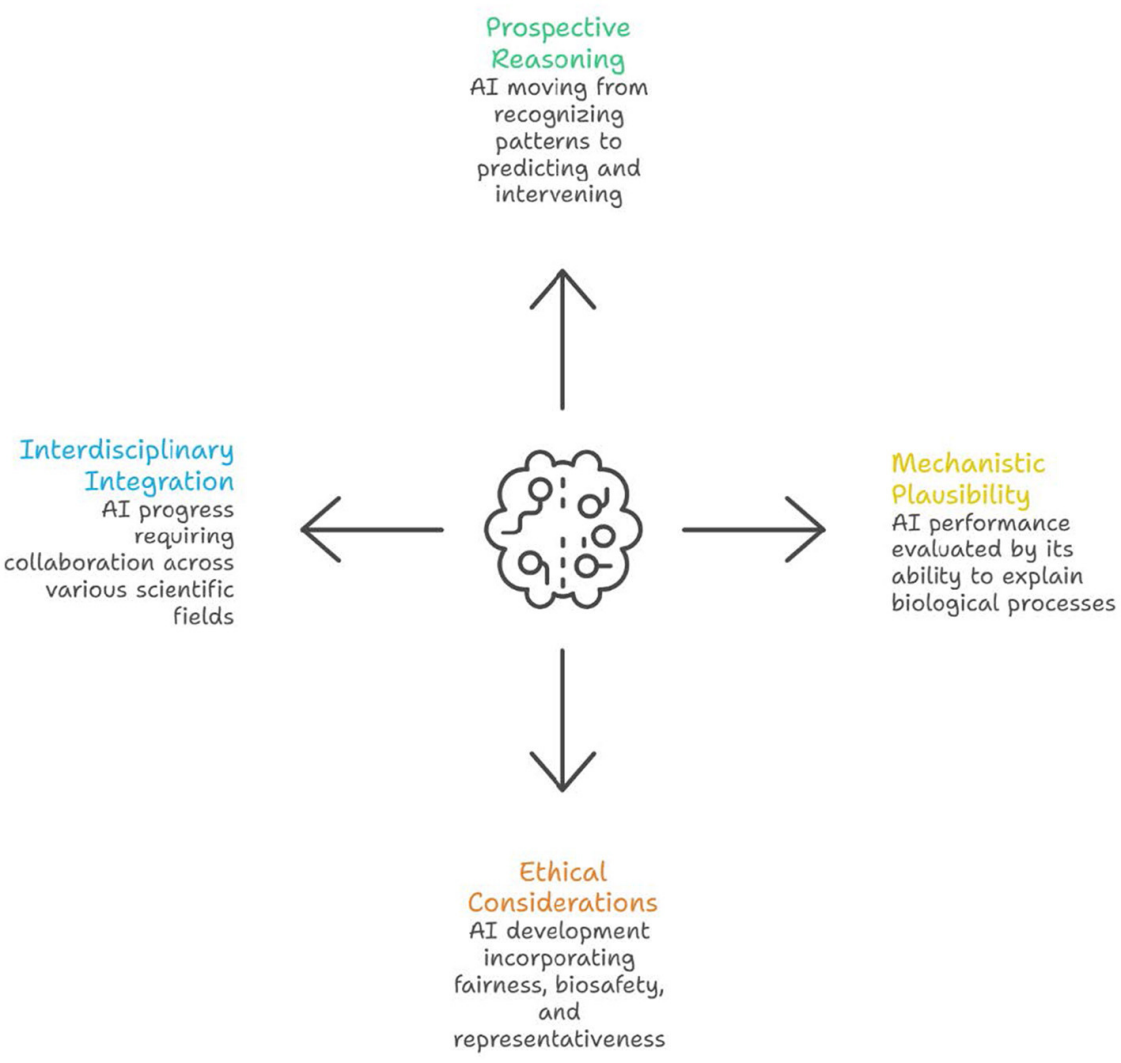
Emerging trajectories of generative AI and large language models in microbial evolution, resistance mechanisms, and antimicrobial drug discovery.

Taken together, as seen from Figure 1, the articles in this Research Topic reveal several broader trajectories. First, microbiological AI is moving from retrospective pattern recognition toward prospective and intervention oriented reasoning. Second, predictive performance alone is increasingly recognized as insufficient unless accompanied by mechanistic plausibility, validation, and interpretability. Third, the field is beginning to recognize that fairness, biosafety, and global representativeness are not external ethical add ons, but intrinsic scientific conditions for trustworthy model development. Finally, these papers collectively demonstrate that future progress in microbial AI will require sustained integration across microbiology, systems biology, causal inference, computational modeling, and public health.

## 3 Conclusion

This Research Topic therefore closes not with a celebration of automation for its own sake, but with a more disciplined proposition. Generative AI and large language models may indeed transform microbial science, but only if their development is anchored in biological realism, methodological transparency, and translational responsibility. Just as importantly, future advances will depend on whether these systems can support robust causal reasoning, reproducible validation, and equitable relevance across diverse microbial, clinical, and global contexts. The contributions assembled here do not exhaust that agenda. They do, however, help define it and offer an important foundation for the next phase of responsible innovation in microbial AI.

